# Editorial: Recent advances in peptide informatics: challenges and opportunities

**DOI:** 10.3389/fbinf.2023.1271932

**Published:** 2023-09-12

**Authors:** Rahul Kumar, Kumardeep Chaudhary, Sandeep Kumar Dhanda

**Affiliations:** ^1^ Department of Biotechnology, Indian Institute of Technology Hyderabad, Kandi, Telangana, India; ^2^ CSIR-Institute of Genomics and Integrative Biology (CSIR-IGIB), New Delhi, India; ^3^ Department of Oncology, St. Jude Children’s Research Hospital, Memphis, TN, United States

**Keywords:** peptide, vaccine candidates, software, database, bioinformatics and computational biology

## Introduction

Peptide informatics is a rapidly growing field that is at the intersection of bioinformatics, chemistry, and biology. Peptides are short chains of amino acids that play important roles in a wide variety of biological processes, such as protein folding, signal transduction, and immune function. Peptide informatics is the use of computational methods to study peptides and their sequence, structure, function, and interactions.

Recent advances in peptide informatics have led to a number of new discoveries and applications. For example, new methods have been developed to predict the structure of peptides, which can be used to design new drugs and therapies. New methods for identifying peptide-protein interactions have also been introduced, which can be used to understand the molecular basis of disease.

## Summary of included articles

Cates et al. developed a web-based resource for exploratory analysis of the data generated from peptide immunoarray experiments. The platform, named “EPIphany”, enables users to upload data files and perform data preprocessing and normalization, followed by intuitive visualization options to gain insights. It is aimed at biological users and provides the greatest opportunity to extract biologically meaningful information from the immunoarray data, e.g., highlighting potential epitopes.

In a quest to predict cross-reactive human protein components with SARS-CoV-2, Moody et al. first predicted the B cell epitopes in SARS-CoV-2 proteins, with the subsequent identification of the association between host proteins, autoimmunity, and cross-reactivity of the predicted epitopes. They isolated more than 100 human proteins that are immunologically likely to be cross-reactive with the COVID-19 pathogen.

Kadam et al. developed a novel algorithm called “AbCPE” to predict the potential antibody class (es) to which a sequential B cell epitope may bind. By utilizing a knowledge base of epitopes that bind to various antibody classes (IgG, IgE, IgA, and IgM), AbCPE derives dipeptide composition, among others, as features for prediction. The authors explored Random Forest and AdaBoost classifiers along with Binary Relevance and Label Powerset techniques. The best performance based on the lowest Hamming loss was achieved using the Binary Relevance model, along with Random Forest and AdaBoost.

Qian et al. discussed the effectiveness of compound-protein interaction (CPI) prediction for drug discovery and proposed enhancements using deep learning methods. The article introduces “CAT-CPI”, a molecular image-based model that combines a Convolutional Neural Network (CNN) and a transformer. CAT-CPI utilizes the former to extract local features from molecular images and employs the latter to capture semantic relationships among these features. By integrating these approaches, CAT-CPI offers an improved framework for accurate CPI prediction based on state-of-the-art performance metrics on diverse datasets and comparisons with other existing tools.

Yang et al. reviewed recent advances in the use of macrocyclic peptides in the context of cancer treatment and the opportunities and challenges they present. Macrocyclic peptides have garnered significant interest by virtue of their moderate size, enhanced selectivity, and strong binding affinities, making them promising therapeutics for targeting dysregulated protein-protein interactions. In addition to three major categories and mechanistic details, this review catalogs those in clinical trials along with FDA-approved candidates. This research opens up new perspectives in the pursuit of effective cancer therapies.

The importance of understanding the conformational ensemble of polypeptides under different experimental conditions for biological applications is discussed in the research article by Tufféry and Derreumaux. The study combines the Debye-Hückel formalism and coarse-grained potential to account for peptide structure prediction in systems beyond neutral pH. Benchmarking with established algorithms shows that this method has advantages in the case of poly-charged peptides and those with missing homologous sequences in public domain repositories. This research sheds light on the improvement of structure prediction techniques for a wide range of polypeptides.

Another review, this time by Bertoline et al., outlined recent groundbreaking advances to predict protein structures, which include the groundbreaking AlphaFold2 and protein language models. Although these tools have limitations, these advances hold promise for revolutionizing structural biology and uncovering the hidden secrets within protein sequences. The authors provide a brief landscape of protein structure prediction approaches, pre- and post-AlphaFold2 release.

## Conclusion

Peptide informatics, despite its challenges, holds immense promise with a multitude of potential applications. The advancement of computational methods dedicated to peptide research will unlock new discoveries and opportunities in the fields of biology, chemistry, and medicine. We invite readers to explore the diverse Research Topic in our Research Topic, which encompasses various applications of peptide informatics, and discover the exciting possibilities it offers.

